# Association between visceral adipose tissue volume, measured using computed tomography, and cardio-metabolic risk factors

**DOI:** 10.1038/s41598-021-04402-5

**Published:** 2022-01-10

**Authors:** Wook Yi, Keunyoung Kim, Myungsoo Im, Soree Ryang, Eun Heui Kim, Mijin Kim, Yun Kyung Jeon, Sang Soo Kim, Bo Hyun Kim, Kyoungjune Pak, In Joo Kim, Seong-Jang Kim

**Affiliations:** 1 Division of Endocrinology and Metabolism, Department of Internal Medicine, Biomedical Research Institute, Pusan National University Hospital, Busan, Republic of Korea; 2 Department of Nuclear Medicine and Biomedical Research Institute, Pusan National University Hospital and School of Medicine, Pusan National University, Busan, Republic of Korea; 3 Department of Nuclear Medicine and Research Institute for Convergence of Biomedical Science and Technology, Yangsan Pusan National University Hospital, Yangsan, Republic of Korea

**Keywords:** Endocrine system and metabolic diseases, Metabolic syndrome

## Abstract

We evaluated the associations between metabolic parameters with visceral adipose tissue (VAT) volume in women with prediabetes or type 2 diabetes (T2DM), and we compared the VAT volume with the VAT area. We enrolled women aged > 20 years with prediabetes or T2DM, who underwent oral glucose tolerance test and whose VAT was evaluated using computed tomography (CT) at our institution between 2017 and 2019. All participants underwent unenhanced spiral CT with a 3-mm slice thickness from the level of the diaphragm to the level of the mid-thigh. The two VAT areas were defined as the free drawn area on the levels of the umbilicus and L2 vertebra. The VAT areas were also manually drawn from the level of the diaphragm to the level of the pelvic floor and were used to calculate the VAT volumes by summing all areas with a slice thickness of 3 mm after setting the attenuation values from −45 to −195 Hounsfield Unit. All metabolic characteristics, except blood pressure, were significantly correlated with the VAT volume. The VAT areas measured at the level of the L2 vertebra and umbilicus were correlated with serum triglyceride, high-density lipoprotein cholesterol, and Framingham steatosis index alone. Multivariable regression analyses revealed that the VAT volume was significantly associated with several metabolic parameters. In conclusion, in women with prediabetes and T2DM, the VAT volume acquired from CT-based calculation has more significant correlations with metabolic risk factors compared with the VAT area.

## Introduction

Overweight and obesity are medical conditions that are characterized by abnormal or excessive fat accumulation. Globally, the prevalence of overweight and obesity has increased significantly in recent decades^1^. Between 2009 and 2015, the prevalence of obesity increased from 29.7% to 32.4%, and the prevalence of obesity-related comorbidities, such as type 2 diabetes (T2DM), hypertension, dyslipidemia, and cardiovascular disease, also steadily increased in the Republic of Korea^2^.

Insulin resistance and β-cell dysfunction play important roles in obesity and obesity-related metabolic diseases; importantly, visceral adipose tissue (VAT) is linked to insulin resistance^3^. Several reports have shown that VAT is more associated with the prevalence of insulin resistance and obesity-related complications than subcutaneous adipose tissue^4–6^. Furthermore, VAT is more strongly associated with metabolic risk factors in women than in men^7^. Thus, the measurement of VAT is required to assess the risk of T2DM and other obesity-related diseases among women.

Currently, computed tomography (CT) is the gold standard for the assessment of VAT, and a single area image at the level of the L4–5 vertebral space is commonly used for its simplicity and to reduce radiation exposure^8^. Previous studies have shown that a single-slice measurement of VAT is strongly correlated with VAT volumes and positively associated with cardio-metabolic risk factors^9,10^. However, the single VAT area of the strongest relation to cardio-metabolic risk factors differ among individuals and vary according to sex and races^10–12^. Therefore, in order to assess the association between VAT and metabolic risk factors, it is necessary to evaluate VAT through a volumetric measurement rather than a single area measurement.

Previously, we showed the correlation between VAT volume and cardio-metabolic risk factors in healthy adults using CT based volumetric measurement^13^. In this study, we evaluated the associations of insulin resistance and cardio-metabolic risk factors with the VAT volume in women with prediabetes or T2DM using CT-based volumetric measurement, and we compared the VAT volume with the VAT area measured using CT-based area measurement.

## Methods

### Study design and population

The study participants included women aged > 45 years with prediabetes or T2DM, who underwent both abdominal CT and oral glucose tolerance tests (OGTT), presented at Pusan National University Hospital (Busan, Republic of Korea), a tertiary medical center, between 2017 and 2019. The electronic records of these patients were retrospectively reviewed and no additional analysis using human tissue samples. Patients were excluded from the study if they had undergone abdominal surgery or were receiving antidiabetic medications. This retrospective study was approved by the Institutional Review Board (IRB) of Pusan National University Hospital, which waived the requirement for written consent (IRB no. 2105-006-102). The study was performed in accordance with the relevant guidelines and regulations.

### Definition of prediabetes and diabetes

Prediabetes and diabetes were defined according to the criteria of the American Diabetes Association. Prediabetes includes impaired fasting glucose (IFG), impaired glucose tolerance (IGT), and hemoglobin A1c (HbA1c) levels of 5.7–6.4%. IFG refers to a fasting plasma glucose level of 100–125 mg/dL, and IGT refers to a 2-h glucose level of 140–199 mg/dL during OGTT (75 g). Diabetes refers to a fasting glucose level of ≥ 126 mg/dL, 2-h glucose level of ≥ 200 mg/dL during OGTT, and HbA1c of ≥ 6.5%^14^.

### Laboratory assessments and metabolic indices

Venous blood samples were taken from all subjects in the morning after 12 h of overnight fasting. Biochemical assays were done using an autoanalyzer (Hitachi 747; Hitachi Corp., Tokyo, Japan). Lipid profiles (total cholesterol (TC; mg/dL), triglycerides (TG; mg/dL), high-density lipoprotein (HDL) cholesterol (mg/dL), and low-density lipoprotein (LDL) cholesterol (mg/dL)), liver function (aspartate aminotransferase (AST) and alanine aminotransferase (ALT)), and renal function (creatinine and blood urea nitrogen) were measured by an enzymatic method. Fasting plasma glucose (mg/dL) was measured by the glucose oxidase method using Synchron LX20 (Beckman Coulter, Fullerton, CA, USA). Plasma insulin (µU/mL) was determined using an enzyme immunoassay (Dainabot, Tokyo, Japan). OGTT was performed to assess glucose metabolism. Each participant drank a 75 g glucose solution, and blood samples were taken at baseline, 30, 60, 90, and 120 min.

Insulin resistance was evaluated using the homeostatic model assessment of insulin resistance (HOMA-IR), calculated as fasting insulin (μU/mL) × fasting glucose/405 (mg/dL); quantitative insulin sensitivity check index (QUICKI), calculated as 1/(log(fasting insulin (μU/mL) + log(fasting glucose (mg/dL)); and Matsuda insulin sensitivity index, calculated as 10,000/(fasting glucose (mg/dL) × fasting insulin (μU/mL) × mean glucose during OGTT (mg/dL) × mean insulin during OGTT (μU/mL))^0.5^ ^15–17^. β-cell dysfunction was determined using homeostatic model assessment of β-cell function (HOMA-β), calculated as 360 × fasting insulin (μU/mL)/(fasting glucose (mg/dL)−63, and insulinogenic index, calculated as the increase in insulin levels from 0 to 30 min divided by the increase in glucose levels from 0 to 30 min^16,18^. Fatty liver was assessed using non-alcoholic fatty liver disease (NAFLD) liver fat score, calculated as −2.89 + 1.18 × metabolic syndrome (yes = 1 / no = 0) + 0.45 × diabetes (yes = 2 / no = 0) + 0.15 × (fasting insulin, mU/L) + 0.04 × AST + 0.94 × AST/ ALT ratio); hepatic steatosis index, calculated as 8 × ALT /AST ratio + body mass index (BMI) (+ 2, if diabetes; + 2, female); and Framingham steatosis index, calculated as − 7.981 + 0.011 × age (years) − 0.146 × sex (female = 1) + 0.173 × BMI (kg/m^2^) + 0.007 × TG (mg/dL) + 0.593 × hypertension (yes = 1, no = 0) + 0.789 × diabetes (yes = 1, no = 0) + 1.1 × ALT/ AST ratio ≥ 1.33 (yes = 1, no = 0)^19–21^.

### Definition of metabolic syndrome

Metabolic syndrome was defined as the presence of three or more of the following components according to the modified National Cholesterol Education Programme Adult Treatment Panel III criteria^22^: (1) abdominal obesity (waist circumference ≥ 85 cm for women, as defined by the Korean Society of Obesity)^23^; (2) hypertriglyceridemia (serum TG concentration of ≥ 150 mg/dL); (3) low HDL cholesterol (serum HDL cholesterol concentration of < 50 mg/dL for women); (4) high blood pressure (systolic blood pressure [SBP] of ≥ 130 mmHg, diastolic blood pressure [DBP] ≥ 85 mmHg, or treatment with antihypertensive agents); and (5) high fasting glucose level (fasting serum glucose level of ≥ 100 mg/dL or previously diagnosed T2DM).

### CT protocol and quantification of abdominal adiposity

Unenhanced spiral CT was performed using Philips Brilliance 16-slice multidetector helical CT scanner (GEMINI TF CT, Philips, Eindhoven, Netherlands) at a voltage of 120 kVp with a slice thickness of 3 mm from the level of the diaphragm to the level of the mid-thigh. The VAT areas were defined as the free drawn area of VAT on the level of the umbilicus and L2 vertebra. The additional VAT areas were manually defined from the level of the diaphragm to the level of the pelvic floor, and these were used in calculating the VAT volumes by setting the attenuation values from −45 to −195 Hounsfield Unit using a CT software (SIEMENS, Syngo CT basic evaluation).

### Statistical analyses

All non-normally distributed variables were expressed as medians and interquartile ranges (IQR; 25–75%). Mann–Whitney *U*-test was used to compare continuous variables in both groups. The *X*^2^-test was used to compare categorical data in both groups. A rank correlation was used during the analysis to show the degree of association between variables. To evaluate the relationship between multiple parameters, we conducted a stepwise multiple linear regression by considering a set of potential variables. Statistical analyses were performed using the MedCalc® version 16.4.3 (MedCalc, Mariakerke, Belgium) software. *P*-values of < 0.05 were indicative of statistical significance.

### Ethics approval and consent to participate

The study complied with the principles of the Declaration of Helsinki. Since no individual patient information is discussed, consent from participants was not needed according to the Institutional Review Board of Pusan National University Hospital.

### Consent for publication

All authors have read the paper and agree that it can be published.

## Results

### Baseline characteristics

A total of 75 patients (median age 61, interquartile range [IQR]: 52.2; 65.0 years) were enrolled. Twenty-one patients (28.0%) were diagnosed with T2DM, and 54 patients (72.0%) were diagnosed with prediabetes. Forty-five patients (60.0%) had metabolic syndrome. Twenty-six patients (34.7%) were treated for hypertension, and the blood pressures of all enrolled patients were relatively well controlled. No patients had decreased renal function. The baseline characteristics details of all patients are summarized in Table 1.

**Table 1:** Baseline Characteristics.

Number of patients	75
Age (years)	61.00 [56.25;65.00]
Systolic blood pressure (mmHg)	122.00 [116.000;129.750]
Diastolic blood pressure (mmHg)	70.00 [63.00;78.00]
**Diabetes status, n (%)**	
Diabetes	21 (28.0)
Prediabetes	54 (72.0)
**Metabolic Syndrome, n (%)**	
Yes	45 (60.0)
No	30 (40.0)
**Medication for Hypertension, n (%)**	
Yes	26 (34.7)
No	49 (65.3)
**Administration of statin, n (%)**	
Yes	43 (57.3)
No	32 (42.7)
Height (cm)	155.6 [152.0;158.7]
Weight (kg)	60.3 [55.0;66.6]
BMI (kg/m^2^)	24.8 [23.4;27.4]
VAT volume (cm^3^)	338.8 [253.8;406.6]
VAT area (L2 level, cm^2^)	10.3 [8.3;12.3]
VAT area (Umbilicus level, cm^2^)	13.0 [10.1;17.0]
Total cholesterol (mg/dL)	176.0 [155.8;194.8]
LDL-cholesterol (mg/dL)	102.1 [88.0;118.3]
HDL-cholesterol (mg/dL)	57.0[50.0;64.5]
Triglyceride (mg/dL)	119.0 [83.5;185.8]
AST (U/L)	22.0 [17.0;28.5]
ALT (U/L)	21.0 [17.0;30.0]
Blood urea nitrogen (mg/dL)	14.5 [12.4;16.6]
Creatinine (mg/dL)	0.67 [0.59;0.72]
Fasting blood glucose (mg/dL)	105 [98;114]
HbA1c (%)	6.03 [5.90;6.30]
HOMA-IR	2.15 [1.69;3.31]
QUICKI	0.340 [0.320;0.352]
Matsuda index	3.70 [2.36;4.62]
HOMA-β	75.9 [53.1;108.9]
Insuliogenic index	0.41 [0.19;0.62]
Hepatic steatosis index	35.8 [33.6;37.9]
NAFLD liver fat score	1.25 [0.40;2.12]
Framinghan steatosis index	− 1.72 [− 2.24; − 0.88]

Values are presented as number (%), or median [interquartile range].

BMI, body mass index; VAT, visceral adipose fat tissue; LDL, low-density lipoprotein; HDL, high-density lipoprotein; AST, aspartate aminotransferase; ALT, alanine aminotransferase; ALP, alkaline phosphatase; HbA1c, hemoglobin A1c; HOMA-IR, homeostatic model assessment of insulin resistance, HOMA-β, homeostatic model assessment of β-cell function; QUICKI, quantitative insulin sensitivity check index; NAFLD, nonalcoholic fatty liver disease.

### The difference in abdominal adiposity indices according to the metabolic syndrome

**Figure 1:**
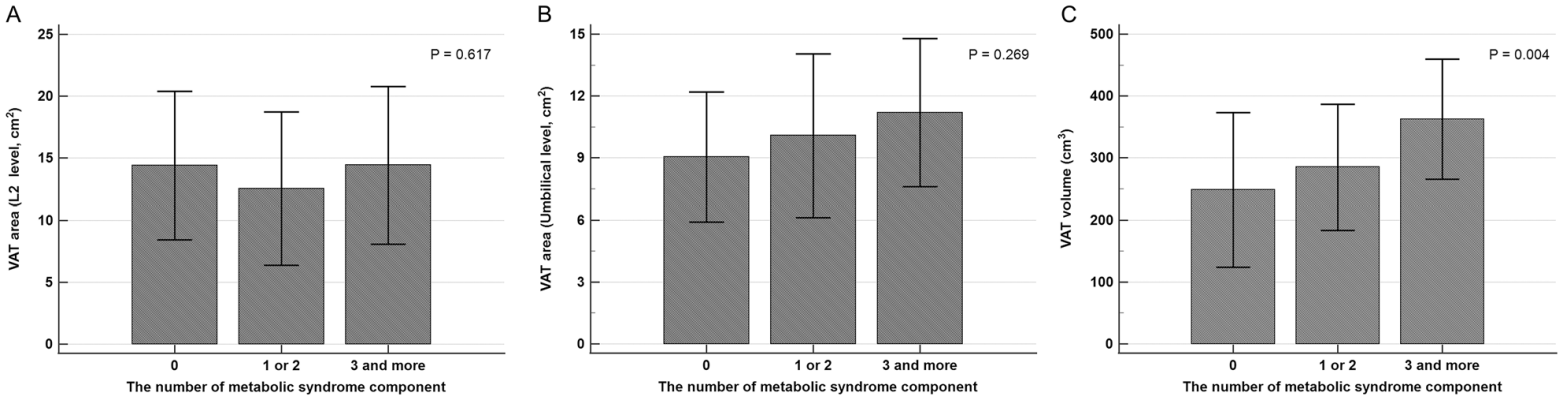
The difference in abdominal visceral adiposity according to the number of metabolic syndrome component. (**A**) Visceral adipose tissue (VAT) area at the level of L2 did not have difference according to the number of metabolic syndrome component. (**B**) VAT area at the level of umbilicus had increasing trend with increasing corresponding number of metabolic syndrome component without statistical significance. (**C**) VAT volume has significantly higher value according to the increasing corresponding number of metabolic syndrome component.

The VAT volume was the lowest in the patients who had no metabolic syndrome compared to the VAT volume in patients with metabolic syndrome, and the VAT volume was increased with the number of correspondences to the metabolic syndrome components with statistical significance. However, according to the number of metabolic syndrome components, the VAT areas were not significantly different (Fig. 1).

### The correlations between abdominal adiposity indices and metabolic characteristics

For all patients, all metabolic characteristics, except blood pressure, were significantly correlated with VAT volume. The VAT areas measured at the level of the L2 vertebra and umbilicus were correlated with the serum levels of TG and HDL cholesterol and Framingham steatosis index. Other metabolic parameters did not show any significant correlation with the VAT areas (Table 2). According to the multivariable regression analyses, the VAT volume showed significant association with several metabolic parameters. The VAT areas showed significant correlations with only serum levels of TG, HDL, and cholesterol, and with the Framinghan steatosis index (Table 3).

**Table 2:** Univariable regression analysis between VAT measurement methods to metabolic risk factors.

		VAT volume (cm^3^)	VAT area (L2 level, cm^2^)	VAT area (Umbilicus level, cm^2^)
Triglyceride	Correlation coefficient	0.309	0.379	0.337
	Significance Level P	0.007	0.008	0.003
HDL-cholesterol	Correlation coefficient	− 0.335	− 0.290	− 0.224
	Significance Level P	0.003	0.012	0.053
Fasting blood glucose	Correlation coefficient	0.292	0.021	0.093
	Significance Level P	0.011	0.856	0.427
Systolic blood pressure	Correlation coefficient	0.035	− 0.000	0.015
	Significance Level P	0.771	0.998	0.900
Diastolic blood pressure	Correlation coefficient	0.082	− 0.174	− 0.073
	Significance Level P	0.494	0.146	0.545
HOMA-IR	Correlation coefficient	0.469	0.019	− 0.022
	Significance Level P	< 0.001	0.869	0.849
QUICKI	Correlation coefficient	− 0.50	− 0.036	0.017
	Significance Level P	< 0.001	0.762	0.886
Matsuda index	Correlation coefficient	− 0.415	0.000	0.052
	Significance Level P	0.003	0.999	0.724
HOMA-β	Correlation coefficient	0.212	− 0.046	− 0.158
	Significance Level P	0.067	0.696	0.176
Insuliogenic index	Correlation coefficient	0.015	0.034	− 0.010
	Significance Level P	0.898	0.774	0.933
Hepatic steatosis index	Correlation coefficient	0.438	0.166	0.155
	Significance Level P	0.0001	0.167	0.197
NAFLD liver fat score	Correlation coefficient	0.496	0.078	0.110
	Significance Level P	< 0.0001	0.519	0.360
Framinghan steatosis index	Correlation coefficient	0.531	0.440	0.413
	Significance Level P	< 0.0001	0.002	0.005

VAT, visceral adipose fat tissue; HDL, high-density lipoprotein; HOMA-IR, homeostatic model assessment of insulin resistance, HOMA-β, homeostatic model assessment of β-cell function; QUICKI, quantitative insulin sensitivity check index; NAFLD, nonalcoholic fatty liver disease.

**Table 3:** Multivariable regression analysis between VAT measurement methods to metabolic risk factors.

		VAT volume (cm^3^)	VAT area (L2 level, cm^2^)	VAT area (Umbilicus level, cm^2^)
Triglyceride	Correlation coefficient	0.164	3.996	
	t	2.184	3.023	
	Significance Level P	0.032	0.004	
HDL-cholesterol	Correlation coefficient	− 0.365	− 0.511	
	t	− 2.570	− 2.054	
	Significance Level P	0.012	0.044	
Fasting blood glucose	Correlation coefficient	0.400		
	t	2.607		
	Significance Level P	0.011		
Systolic blood pressure	Correlation coefficient			
	t			
	Significance Level P			
Diastolic blood pressure	Correlation coefficient			
	t			
	Significance Level P			
HOMA-IR	Correlation coefficient	0.514		
	t	4.541		
	Significance Level P	< 0.001		
QUICKI	Correlation coefficient	0.038		
	t	− 4.937		
	Significance Level P	< 0.001		
Matsuda index	Correlation coefficient	− 0.060		
	t	− 2.763		
	Significance Level P	0.008		
HOMA-β	Correlation coefficient			
	t			
	Significance Level P			
Insuliogenic index	Correlation coefficient			
	t			
	Significance Level P			
Hepatic steatosis index	Correlation coefficient	0.012		
	t	4.043		
	Significance Level P	< 0.001		
NAFLD liver fat score	Correlation coefficient	0.056		
	t	4.746		
	Significance Level P	< 0.001		
Framinghan steatosis index	Correlation coefficient	0.004	0.512	
	t	4.591	3.427	
	Significance Level P	< 0.001	0.001	

VAT, visceral adipose fat tissue; HDL, high-density lipoprotein; HOMA-IR, homeostatic model assessment of insulin resistance, HOMA-β, homeostatic model assessment of β-cell function; QUICKI, quantitative insulin sensitivity check index; NAFLD, nonalcoholic fatty liver disease.

## Discussion

In this cross-sectional study, we showed that the VAT volume was associated with insulin resistance, cardio-metabolic risk factors, and hepatic steatosis in women with prediabetes and in those with T2DM. Compared with the VAT area at the L2 vertebra, the VAT volume at the umbilicus had a stronger correlation. Furthermore, insulin resistance and fatty liver index were significantly associated with the VAT volume.

Irlbeck et al. previously showed that the VAT volume was best correlated with cardio-metabolic risk factors in the Framingham Heart Study, and the VAT area had a similar association with VAT volume^10^. The result of this cross-sectional study is consistent with a previous report on lipid profiles (TG and HDL cholesterol), although fasting blood glucose was associated with the VAT volume alone, and blood pressure was not significantly associated with both the VAT volumes and VAT areas. Overall, 26 patients (34.7%) were already taking antihypertensive medications, and both SBP and DBP were in the normal range in most patients in this study (SBP range from 116 to 129 mmHg, DBP range from 63 to 78 mmHg). Thus, the prescriptions of hypertensive medication and blood pressure value affected the results of this study. Furthermore, the association with metabolic syndrome, which is a cluster of cardio-metabolic risk factors, has been reported in many studies. According to a longitudinal cohort study in the Republic of Korea, VAT was significantly associated with the incidence of metabolic syndrome and incidence of each component of metabolic syndrome^24^. In addition, other longitudinal studies containing participants of various ethnicities showed the relationship between VAT and metabolic syndrome^25,26^. Increased VAT volume was correlated with the number of metabolic syndrome components in this study, although the VAT areas were not significantly correlated.

The adipose tissue releases adipokines, hormones, and free fatty acid that modulate glucose and lipid metabolism, insulin sensitivity, and inflammation; thus, excessive secretion of these molecules can contribute to insulin resistance and hepatic steatosis^27–29^. In addition to liver inflammation, the size of liposomes in hepatocytes increases with excess adiposity and results in hepatic steatosis^30^. Thus, we compared the VAT volumes and VAT areas using insulin resistance, β-cell dysfunction, and hepatic steatosis in women with prediabetes and in those with T2DM. Consistent with the findings of previous studies, the VAT volume and hepatic steatosis index were correlated in this study^29,31^. Interestingly, the β-cell dysfunction indexes were not significantly associated with the VAT volume, although insulin resistance indexes and hepatic steatosis indexes were significantly related to the VAT volume. Recently, Wang et al. showed that insulin resistance had a stronger association with T2DM than it did with β-cell dysfunction, especially in obese patients^32^. Furthermore, women were found to have higher capacities for insulin secretion and incretin responses than men^33^. For these reasons, insulin sensitivity indexes were not significantly associated with the VAT volume in this study.

Due to radiation exposure, CT based VAT measurement has been used in a limited condition of cross-sectional study^8^. However, abdominal CT scan has been frequently performed and has dramatically increased over the past several decades for health screening and for other various medical conditions for diagnosis and follow-up of diseases affecting abdominal organs. For the patients with diabetes or predibetes, abdominal CT could be used to be ruled out any kinds of pancreatic tumor, especially in patients with sudden onset of diabetes^33,34^. In addition, there are several studies to approach the in vivo pancreatic endocrine function using CT^35–38^. Therefore, much of the patients already had abdominal CT in the current study and the CT based VAT measurement could provide more specific additional metabolic information without additional radiation exposure.

This study has several limitations. First, this is a cross sectional study with a retrospective design and a small study, thereby precluding inferences of causality or temporality. Moreover, there is a probability of selection bias because all the patients were from a single center. However, the results of the correlation between the VAT volume and cardio-metabolic risk factors in this study were consistent with the results of previous reports^10^. Second limitation is that this study was conducted among women alone. Sex differences in body fat distribution and metabolic homeostasis are well established in the literature. Women have a lower amount of VAT, but a greater risk of obesity and a higher prevalence of metabolic syndrome than men^39,40^. Furthermore, endocrine function of VAT differs by sex. Women have higher leptin and adiponectin levels; thus, these hormones may be causes of sex differences in insulin sensitivity and metabolism^41,42^. Therefore, we evaluated the relationship between VAT and cardio-metabolic risk factors in each sex. The number of men in the study population was too small to assess the correlation between VAT and cardio-metabolic risk factors, because of which we discussed these relations in women alone. Finally, the population of this study comprised relatively elderly patients (median age 61.0, IQR 52.265.6) of Korean population. Thus, generalisation of our results to younger population and other ethnical group should be made with caution.

## Conclusions

We showed that the VAT volume, according to CT-based volumetric measurement, was associated with insulin resistance and metabolic risk factors and correlated more compared with the VAT area in women with prediabetes and in those with T2DM. Therefore, CT-based VAT volumetric measurements would be useful methods for researcher evaluating the association VAT and insulin resistance in metabolic high risk population.

## Data Availability

Data are available on request.

## Abbreviations

T2DM: Type 2 diabetes
VAT: Visceral adipose tissue
CT: Computed tomography
OGTT: Oral glucose tolerance tests
IFG: Impaired fasting glucose
IGT: Impaired glucose tolerance
TC: Total cholesterol
TG: Triglycerides
HDL: High-density lipoprotein
LDL: Low-density lipoprotein
AST: Aspartate Aminotransferase
ALT: Alanine aminotransferase
HOMA-IR: Homeostatic model assessment of insulin resistance
QUICKI: Quantitative insulin sensitivity check index
HOMA-β: Homeostatic model assessment of β-cell function
NAFLD: Non-alcoholic fatty liver disease
BMI: Body mass index
SBP: Systolic blood pressure
DBP: Diastolic blood pressure
IQR: Interquartile ranges
