# Author Correction: Association between visceral adipose tissue volume, measured using computed tomography, and cardio-metabolic risk factors

**DOI:** 10.1038/s41598-023-38677-7

**Published:** 2023-08-07

**Authors:** Wook Yi, Keunyoung Kim, Myungsoo Im, Soree Ryang, Eun Heui Kim, Mijin Kim, Yun Kyung Jeon, Sang Soo Kim, Bo Hyun Kim, Kyoungjune Pak, In Joo Kim, Seong-Jang Kim

**Affiliations:** 1 Division of Endocrinology and Metabolism, Department of Internal Medicine, Biomedical Research Institute, Pusan National University Hospital, Busan, Republic of Korea; 2 Department of Nuclear Medicine and Biomedical Research Institute, Pusan National University Hospital and School of Medicine, Pusan National University, Busan, Republic of Korea; 3 Department of Nuclear Medicine and Research Institute for Convergence of Biomedical Science and Technology, Yangsan Pusan National University Hospital, Yangsan, Republic of Korea

Correction to: *Scientific Reports* 10.1038/s41598-021-04402-5, published online 10 January 2022

In the original version of this Article, Affiliation 2 was incorrectly given as ‘Department of Nuclear Medicine and Biomedical Research Institute, Pusan National University Hospital, 179, Gudeok-ro, Seo-gu, Busan, Republic of Korea’. The correct affiliation is:

Department of Nuclear Medicine and Biomedical Research Institute, Pusan National University Hospital and School of Medicine, Pusan National University, Busan, Republic of Korea

The original Article has been corrected.

